# Gut microbiota turn up the heat after bariatric surgery

**DOI:** 10.15698/cst2023.10.290

**Published:** 2023-08-15

**Authors:** Mohammed K. Hankir

**Affiliations:** 1 Department of General, Visceral, Transplantation, Vascular and Pediatric Surgery, University Hospital Wuerzburg, Wuerzburg, Germany.

**Keywords:** vertical sleeve gastrectomy, Roux-en-Y gastric bypass, gut microbiota, thermogenesis, obesity, Type 2 diabetes

## Abstract

Bariatric surgeries like vertical sleeve gastrectomy (VSG) and Roux-en-Y gastric bypass (RYGB) cause well-established shifts in the gut microbiota, but how this contributes to their unique metabolic benefits is poorly understood. Jin *et al* and Yadav *et al* now provide two complementary lines of evidence suggesting that gut microbiota-derived metabolites after VSG and RYGB activate thermogenesis in fat through distinct mechanisms, to in turn promote weight loss and/or improvements in glycemic control.

Despite the implementation of numerous weight loss initiatives by various governments, the global prevalence of obesity continues to rise [1]. This not only takes an often dismissed personal toll on many individuals living with obesity [2], but also represents an enormous burden to healthcare systems [3] and is a leading preventable cause of death [1]. Of the treatments that are currently available for severe obesity, bariatric surgeries such as vertical sleeve gastrectomy (VSG) and Roux-en-Y gastric bypass (RYGB) remain the most effective [4]. These surgeries not only cause unparalelled levels of weight loss (up to 25-35%) [5], but also induce a host of other metabolic benefits including remission of the type 2 diabetes [6] and amelioration of the fatty liver disease [7] that usually accompany obesity. A number of mechanisms have been proposed to contribute to these favourable metabolic outcomes, most notably the rise in circulating gut hormones like glucacon-like peptide 1 (GLP-1) [8]. There are also well-established shifts in the gut microbiota after bariatric surgery, although this appears to be more pronounced for RYGB than for VSG likely due to the differences in anatomical configurations between the two procedures [9]. Interestingly, while gut hormone preperations including the stable GLP-1 analogue semaglutide cause weight loss approaching the level of bariatric surgery (up to 10-20%) [10], GLP-1 receptor signalling seems to be dispensable for the metabolic benefits of both VSG [11] and RYGB [12], at least in rodent models. Therefore, the hunt is still very much on for the identification of factors that contribute to the weight loss and improved glycemic control associated with bariatric surgery, as this will undoubtedly aid in the discovery of novel anti-obesity and type 2 diabetes drugs.

Since its rediscovery in adult humans, research into thermogenic fat has increased tremendously [13]. This is because activation of thermogenesis in fat either by cold exposure or by pharmacological means has long been known to promote metabloic health in rodents, and it is becoming increasingly evident that it does so in the clinical setting, too [13]. While both gut microbiota and bariatric surgery have been shown to activate thermogenesis in fat [14], their connection has, until recently, not been addressed. Two new studies by Jin *et al* [15] and Yadav *et al* [16] now provide causal evidence, using complementary approaches, that VSG and RYGB activate thermogenesis in fat through distinct microbiota-dependent mechanisms, to in turn promote metabolic health.

In the first study by Jin *et al* [15] VSG and sham surgery were performed on diet-induced obese mice. The authors found that VSG-operated mice displayed reduced body weight associated with increased oxygen consumption compared with sham-operated mice. Interestingly, this was not associated with lasting reductions in food intake in VSG-operated mice, reiterating how rodent models of bariatric surgery achieve weight loss predominantly through increasing energy expenditure rather than through decreasing energy intake, which is the opposite to the case in humans [17]. When the authors analysed the 3 major fat depots in mice, they found that the thermogenic mitochondrial molecule uncoupling protein 1 (UCP1) was increased only in subcutaneous white fat and not in epididymal white fat or interscapular brown fat. Because the sympathetic nervous system is an important mediator of thermogenesis, the authors then denervated subcutaneous white fat by injecting the neurotoxin 6-hydroxydopamine locally into this depot. Unexpectedly, this had minimal impact on the efficacy of VSG to exert its metabolic benefits and to increase UCP1 expression in subcutaneous white fat. At this point, it should be stated that these findings contrast sharply with the impact of VSG on diet-induced obese rats, in which it was shown that sympathetic nervous system innervation of brown fat is essential for the reductions in body weight and improvements in glycemic control postoperatively [18], again reiterating species differences in the mechanisms of bariatric surgery.

Jin *et al* [15] then reasoned that gut microbiota-derived products could mediate the activation of thermogenesis in subcutaneous white fat after VSG. Indeed, the authors could confirm that VSG increased the fecal abundance of *Firmicutes* and decreased the fecal abundance of *Bacteriodetes*, associated with stabilization of the intestinal epithelial barrier. Mass spectrometry analysis then revealed that 3 metabolites were increased in the feces of VSG-operated mice: licoricidin, muramic acid, and 3-hydroxybutyryl carnitine. Moreover, fecal licoricidin not only negatively correlated with various gut microbiota known to promote metabolic disease, but circulating licoricidin levels were doubled in VSG-operated mice suggesting that it could communicate directly with subcutaneous white fat. To formally test if gut microbiota-derived products contribute to thermogenesis in subcutaneous white fat after VSG, mice were treated with a broad spectrum antibiotic cocktail via their drinking water for 2 weeks. Strikingly, this not only prevented the weight loss and metabolic benefits associated with VSG similar to a previous study in diet-induced obese mice [19], but it also prevented the increase in oxygen consumption and subcutaneous white fat UCP1 expression as well as the increase in circulating licoricidin. However, this relatively unspecific approach does not reveal which gut microbiota produce and release licoricidin, which is an isoflavenoid normally found in dietary licorice, nor does it prove that licoricidin *per se* contributes to the activation of thermogenesis in subcutaneous white fat after VSG.

Next, Jin *et al* [15] determined if licoricidin given alone via oral gavage can at least recapitulate the metabolic benefits of VSG. Remarkably, licoricidin when admistered via this route prevented body weight gain, improved glycemic control, increased oxygen consumption and increased UCP1 expression in subcutaneous white fat. To dissect the molecular mechanism by which licoricidin activates thermogenesis in subcutaneous white fat, cell culture experiments were performed. Like the sympathetic nervous system, licoricidin recruited the protein kinase A (PKA) signalling pathway in primary subcutaneous white adipocytes as shown through phosphoblots of PKA substrates including cyclic AMP response element binding protein (CREB). Interestingly, the tripling of intacellular cAMP levels by licoricidin treatment did not appear to be due to the inhibition of phosphodiesterases 3 and 4 which is known to promote thermogenesis in epididymal white fat [20, 21] and subcutaneous white fat [22], respectively. Instead, through a combination of confocal microscopy on primary subcutaneous white adipocytes, pull-down assays on beta-3 adrenergic receptor-overexpressing human embryonic kidney (HEK) cells and bioinformatic analysis, licoricidin was shown to bind to transmembrane 3 (TM3), TM6 and TM7 of the beta-3 adrenergic receptor. Moreover, the induction of UCP1 protein in primary subcutaneous white adipocytes by licoricidin was abrogated in cells with knockdown of the beta-3 adrenergic receptor, providing strong evidence that licoricidin promotes thermogenesis in subcutaneous white fat by positively modulating beta-3 adrenergic receptor activity. Finally, to determine if local licoricidin action in subcutaneous white fat is sufficient to recapitulate the effects of systemic licoricidin, it was injected at lower doses directly into this depot. This targetted licoricidin treatment had overlapping metabolic effects with systemic licoricidin treatment, although experiments in PR domain containing 16 (PRDM16)-deficient mice, which are incapable of showing themogenesis in subcutaneous white fat in response to beta-3 adrenergic receptor agonist treatment [23], are needed to determine to what extent activation of thermogenesis in subcutaneous white fat by licoricidin contributes to the metabolic benefits of VSG as well as systemic licoricidin treatment.

In the other study by Yadav *et al* [16] stool samples were collected from 4 patients with morbid obesity before and 1-6 months after RYGB, and subsequently transferred to germ-free mice. What sets the approach of Yadav *et al* [16] apart from similar studies is that samples before and after RYGB from the same individual patient were orally gavaged into recipient germ-free mice in a longitudinal design rather than in a cross-sectional design, which preserves the inter-individual variability in the response to bariatric sugery. Another notable detail of the study of Yadav *et al* [16] is that the germ-free mice in their study were rendered obese on a sterile and costly Western-style diet prior to receiving stool samples from patients. Perhaps equally as important is the delayed timepoint after fecal microbiota transfer (12 weeks) that recipient germ-free mice were subjected to metabolic phenotyping, allowing sufficient time for the transferred fecal microbiota to expand and colonize the gastrointestinal tract and exert metabolic effects. In this way, Yadav *et al* [16] found that mice receiving post-RYGB feces have improved glycemic control and increased oxygen consumption compared to mice receiving pre-RYGB feces. Notably, this was independent of any effects on food intake and body weight, suggesting that post-RYGB fecal microbiota have little impact on regulating energy homeostasis. The authors then performed ^18^F-fluorodeoxyglucose positron emission tomography (^18^F-FDG PET) imaging experiments to determine if brown fat contributes to increased energy expenditure in mice receiving post-RYGB fecal microbiota. This revealed that brown fat ^18^F-FDG uptake in response to overnight cold exposure, the natural stimulus for brown fat, almost doubles in mice receiving post-RYGB feces compared with mice receiving pre-RYGB feces. Accordingly, brown fat UCP1 protein expression was also markedly enhanced.

Considering that the post-RYGB feces-treated mice showed enhanced insulin sensitivity in the absence of any differences in body weight, it would have been interesting to determine if insulin increases brown fat ^18^F-FDG uptake. This could have provided evidence that post-RYGB microbiota enhance blood glucose clearance in response to insulin treatment through using brown fat as a glucose sink. It would have also been interesting to determine if the patients themselves showed increased oxygen consumption and brown fat ^18^F-FDG uptake after RYGB, which would have provided the key evidence that this beneficial metabolic change in the host is potentially gut microbiota-mediated and is transmissable to germ-free mice.

Finally, after showing that differences in patient fecal microbiota after RYGB can be transferred to germ-free mice, such as an increase in Akkermansia municiphila, Yadav *et al* performed metabolomic analyses of their feces. This revealed that thermogenic molecules like the short chain fatty acid butyrate [24, 25] and acylcarnitine [26] were increased in mice receiving post-RYGB fecal microbiota along with tryptophan metabolites, while various amino acids including the branched chain ones valine, leucine and isoleucine which are thought to induce insulin resistance [27] were reduced although this has been shown not to be essential for the improvements in glycemic control after bariatric surgery [28]. Considering that for brown fat to be affected by these metabolites they need to first reach the circulation, it would have been interesting to perform metabolomics on systemic blood as well as feces like in the study of Jin *et al* [15]. It would have also been interesting to determine if butyrate does indeed contribute to activation of brown fat themogenesis and improved glycemic control by post-RYGB fecal microbiota through inhibiting monocaroxylate transporter 1 (MCT1) which has been shown to mediate butyrate's thermogenic actions on brown adipocytes [25].

While we are witnessing the dawn of a new era for obesity pharmacotherapy with gut hormone preperations causing unprecendented levels of weight loss, bariatric surgery remains the most effective [4]. The high quality studies of Jin *et al* [15] and Yadav *et al* [16] are groundbreaking in the field as they provide causal evidence that the well-established shifts in gut microbiota after bariatric surgery contribute to improved metablic outcomes by activating thermogenesis in fat, possibly through microbial metabolites such as licoricidin and butyrate (**Figure 1**). Notably, changes in bile acid metabolism by gut microbiota after VSG leads to the generation of cholic acid-7-sulfate in the liver and its accumulation in the gut [29], which has been shown to promote metabolic health in diet-induced obese mice through increasing endogenous GLP-1 release [30]. Thus, understanding the molecular and cellular mechanisms behind how bariatric surgery favourably impacts various metabolic tissues provides a treasure trove for identifying novel factors to treat obesity and type 2 diabetes.

**Figure 1:**
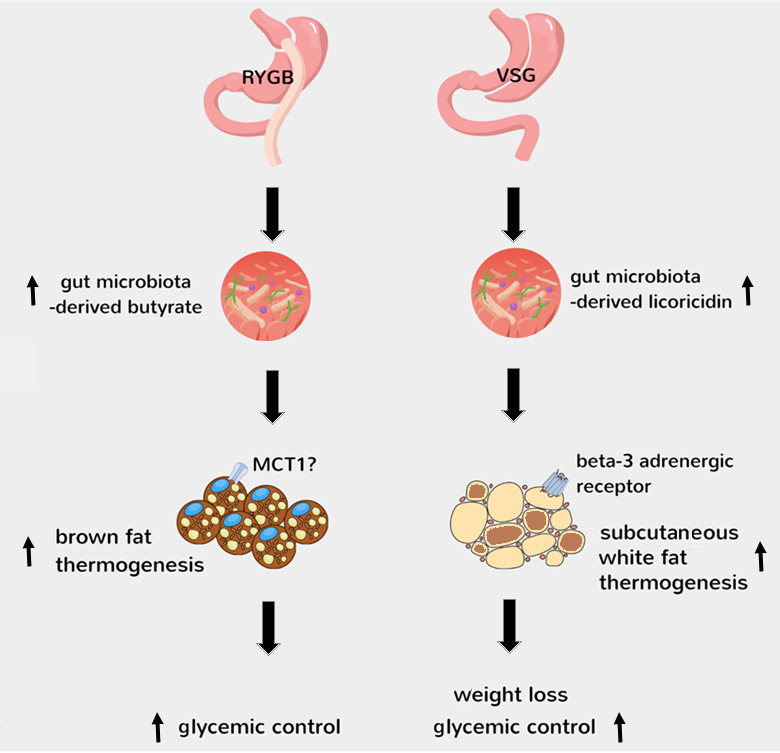
FIGURE 1: Mechanisms behind activation of thermogenesis by gut microbiota after bariatric surgery. The findings of Jin *et al* [15] and Yadav *et al* [16] demonstrate that the shifts in gut microbiota after RYGB and VSG lead to increased gut levels of butyrate and licoricidin, respectively. This then leads to activation of brown fat thermogenesis after RYGB, possibly via a mechanism involving monocarboxlate transporter 1 (MCT1)-mediated transport of circulating butyrate into brown adipocytes [25], and activation of subcutaneous white fat thermogenesis after VSG, possibly via a mechanism involving the positive modulation of beta-3 adrenergic receptor in white adipocytes by increased circulating licoricidin [15].

The findings of Jin *et al* [15] and Yadav *et al* [16] demonstrate that the shifts in gut microbiota after RYGB and VSG lead to increased gut levels of butyrate and licoricidin, respectively. This then leads to activation of brown fat thermogenesis after RYGB, possibly via a mechanism involving monocarboxlate transporter 1 (MCT1)-mediated transport of circulating butyrate into brown adipocytes [25], and activation of subcutaneous white fat thermogenesis after VSG, possibly via a mechanism involving the positive modulation of beta-3 adrenergic receptor in white adipocytes by increased circulating licoricidin [15].
